# Clinical and Immunological Perspectives on the Nasal Microbiome’s Role in Olfactory Function and Dysfunction

**DOI:** 10.3390/microorganisms14010234

**Published:** 2026-01-20

**Authors:** Farwa Mukhtar, Antonio Guarnieri, Maria Di Naro, Daria Nicolosi, Natasha Brancazio, Attilio Varricchio, Antonio Varricchio, Muhammad Zubair, Tamar Didbaridze, Giulio Petronio Petronio, Roberto Di Marco

**Affiliations:** 1 Department of Medicina e Scienze della Salute “V. Tiberio”, Università degli Studi del Molise, 86100 Campobasso, Molise, Italy; f.mukhtar@studenti.unimol.it (F.M.); a.guarnieri@studenti.unimol.it (A.G.); m.dinaro@studenti.unimol.it (M.D.N.); n.brancazio@studenti.unimol.it (N.B.); attilio.varricchio@unimol.it (A.V.); 2 Department of Drug and Health Sciences, Università degli Studi di Catania, 95125 Catania, Sicily, Italy; daria.nicolosi@unict.it (D.N.); roberto.dimarco@unict.it (R.D.M.); 3 Department of Otolaryngology, Azienda Ospedaliera Università di Padova, 35128 Padova, Veneto, Italy; antonio.varricchio@studenti.unipd.it; 4 Department of Bioinformatics and Biotechnology, Government College University, Faisalabad 38000, Pakistan; muhammad.zubair1751@gmail.com; 5 Department of Microbiology, Tbilisi State Medical University, 0186 Tbilisi, Georgia; t.didbaridze@tsmu.edu

**Keywords:** nasal microbiome, olfactory function, chronic rhinosinusitis, neuroinflammation, nasal–brain axis

## Abstract

The nasal microbiome represents a complex and dynamic microbial ecosystem that contributes to mucosal defense, epithelial homeostasis, immune regulation, and olfactory function. Increasing evidence indicates that this microbial community actively interacts with host physiology, while alterations in its composition are associated with chronic inflammation, oxidative stress, and olfactory impairment. Such changes have been reported in conditions including chronic rhinosinusitis, allergic rhinitis, and post-viral anosmia. Beyond local effects, chronic nasal inflammation has been hypothesized to influence neuroinflammatory processes and protein aggregation pathways involving α-synuclein and tau, potentially linking nasal microbial imbalance to neurodegenerative mechanisms. However, current evidence remains largely indirect and does not support a causal relationship. This narrative review summarizes current clinical and immunological evidence on the role of the nasal microbiome in olfactory function and dysfunction, highlighting limitations of existing studies and outlining future research directions.

## 1. Introduction

The human sense of smell plays a fundamental role in everyday life. Beyond influencing flavor perception and environmental awareness, it directly affects emotions, behavior, and overall quality of life [1,2]. In addition to its sensory function, the olfactory system acts as an early warning mechanism against environmental toxins and pathogens, thereby contributing to survival and health [3]. Olfactory receptor neurons, which promote sustentacular cells and basal progenitors, form the olfactory epithelium (OE), present in the superior nasal cavity, and can be regularly replaced throughout life [4]. These sensory nerve fibers synapse with the olfactory bulb via the cribriform plate and form a direct anatomical and functional connection between the nasal mucosa and the central nervous system [5]. It is now recognized that the nasal cavity is not a sterile environment but a complex microbial ecosystem. The nasal microbiome plays a crucial role in mucosal homeostasis and immune regulation [6]. The predominant bacterial phyla are Firmicutes, Actinobacteria, Proteobacteria, and Bacteroidetes, with common genera such as *Corynebacterium*, *Staphylococcus*, *Moraxella*, and *Dolosigranulum* being the significant representatives of the healthy nasal microbiota [7]. This microbial community serves as a biological barrier that limits pathogen colonization and regulates epithelial and immune activity [8].

Nasal microbiome composition depends on age, environment, and host immunity. In the early life stage, maternal contact and environmental exposure influence the colonization patterns that are essential to the maturation of the immune system [9]. Microbial diversity in adults reflect lifestyle, occupation, and environmental pollutants, and poor microbial diversity in older people is frequently accompanied by chronic inflammation and dysfunction of the epithelium [10]. Such changes in the balance of microbes may have a profound effect on the physiology of the nose and predisposition to infection. Commensal microorganisms support immune regulation and epithelial defense in the nasal cavity by producing antimicrobial peptides (AMPs) and metabolites such as short-chain fatty acids (SCFAs) [11]. These metabolites control the mucosal pH, epithelial tight-junction integrity, and local immune signaling using G protein-coupled and Toll-like receptors (TLRs) [12]. Homeostatic microbial cues promote olfactory receptor expression and sensory neuron regeneration. In contrast, dysbiosis stimulates inflammatory cytokine release and disrupts olfactory signal transduction [13].

Viruses have been shown to disrupt the nasal microbial community, or dysbiosis, which has been associated with different diseases, such as chronic rhinosinusitis (CRS), allergic rhinitis, and olfactory dysfunction (OD), caused by viruses [14]. In CRS, lower prevalence of advantageous taxa, including *Corynebacterium* and *Dolosigranulum*, and amplified opportunistic pathogenic taxa, such as *Staphylococcus aureus* and *Pseudomonas aeruginosa*, are factors of ongoing mucosal swelling [15]. Such modifications in microbes result in heightened synthesis of pro-inflammatory cytokines like IL-6 and TNF-α, which disrupt the fixing of the epithelia and turnover of olfactory neurons [16].

The nasal microbiome and olfactory health also depend on environmental factors. Air pollution, PM, and tobacco smoke exposure lower the diversity of microorganisms and contribute to oxidative stress in the mucosa [17]. Additional impacts on the stability of the commensal population are antibiotic use and diet or humidity modifications affecting immune responses and epithelial functions [18]. Notably, the nasal cavity is a special point of contact between microbial, immune, and neural responses. These systems need to communicate with each other to preserve olfactory functions (OF) and mucosal well-being [19]. This interface may be prone to chronic inflammation. In some contexts, inflammatory processes may extend beyond the nasal epithelium and engage neuroinflammatory pathways relevant to neurodegenerative diseases such as Parkinson’s disease (PD) and Alzheimer’s disease (AD) [20].

The fact that early olfaction loss is seen in these pathologies is a contributor to the hypothesis that nasal dysbiosis and mucosal inflammation might be antecedents of central nervous pathology [21]. Nasal microbiome in this regard is a biological mediator, as well as a possible biomarker of health and disease. Knowledge of its composition and its role is critical to understanding the processes associated with the linkage of mucosal immunity, sensory functioning, and neuroinflammation [22].

The purpose of this narrative mini-review is thus to provide an overview of the existing body of knowledge in clinical and immunological applications of the nasal microbiome in OD and OF. The importance of microbial diversity, epithelial–immune crosstalk, and inflammatory sensory loss in CRS, allergic rhinitis, and viral infections is stressed. The review further examines current knowledge of the nasal–brain axis with regard to the potential role of chronic dysbiosis and immune activation in causing neuropathological alterations.

The literature search was conducted using PubMed, Scopus, and Web of Science to identify studies addressing the nasal microbiome in relation to olfactory function, immunity, and neurological conditions. Searches were performed up to 2024 using combinations of relevant keywords (e.g., nasal microbiome, olfactory dysfunction, chronic rhinosinusitis, and neurodegeneration). Peer-reviewed human and animal studies providing information on microbiome–olfactory interactions were preferentially considered, while editorials, opinion pieces, and studies unrelated to the nasal microbiome were excluded. Articles were screened based on titles and abstracts, followed by full-text evaluation of relevant studies. The final selection focused on studies of high relevance and methodological quality, consistent with the narrative scope of the review.

## 2. The Nasal Microbiome and Its Composition

The nasal cavity has a dynamic microbiome, which is dependent on the region, the exposure to the environment, and the physiology of the host. These microbial communities are involved in functions that are important in immune tolerance, maintenance of barriers, and defense against pathogens [23,24,25]. Equilibrium in the microbiome is also associated with olfactory perception, and dysbiosis can interfere with mucosal–neuronal communication [26,27].

### 2.1. Microbial Diversity and Spatial Distribution

The nasal cavity has distinct anatomical regions that form specific ecological niches that influence microbial colonization [28]. *Staphylococcus epidermidis*, *Corynebacterium accolens*, and *Cutibacterium acnes* can be found in the front nares and are in stable consortia to defend the host by competitive exclusion of pathogens [29]. *Moraxella*, *Haemophilus*, and *Streptococcus* are usually found in the middle meatus and the nasopharyngeal areas [30].

Microbial communities have unique spatial distributions and clinical implications of the anatomical niches of the nasal cavity are summarized in Table 1. Protective commensals predominate the anterior nares including *S. epidermidis* and *Corynebacterium*, which play a role in protecting the epithelium and can be involved in the health of the olfactory system due to their ability to influence the immune response [31]. Conversely, opportunistic genera such as *Moraxella*, *Klebsiella*, and *Escherichia* are more commonly found in the nasopharyngeal region and are more likely to cause respiratory infections and inflammatory reactions, leading to the loss of olfaction [32].

Table 1 draws attention to the occupancy of particular taxa in the defined layers of the epithelium of the vestibular to nasopharyngeal area and outlines the possible pathogenic or probiotic functions. One example is *Dolosigranulum pigrum* and *Bifidobacterium*, which are also becoming recognized as useful residents known to stabilize the mucosal environment, while *Lawsonella clevelandensis* and *Neisseriaceae* are new taxa whose olfactory roles remain unclear [35,37,38]. These space distributions represent that microbial composition and regionalization to an anatomical site are vital factors of nasal immune tone and sensory activity. Such patterns of microbes are affected by various host factors such as age, immune system maturity, hormonal balance, and exposure to the environment [42]. During their delivery and initial interactions, newborns obtain their nasal microbiota through maternal sources and form a baseline of immune development [43]. Microbial diversity in adults is related to workplace and lifestyle exposures, but in older patients, low diversity is routinely linked to an increased occurrence of pathogenic species and inflammatory diseases [36].

All these observations highlight the dynamic homeostasis between the nasal microbiome and host physiology. Any disturbance of this balance, by infection, the use of antimicrobials, or environmental disputes, can make people vulnerable to inflammatory and olfactory diseases [37].

### 2.2. Determinants of Microbial Stability

The stability of microbes in the nasal cavity is determined by host immunity, mucosal secretions, and interactions between the bacteria. *S. epidermidis*, which is a commensal bacterium, releases proteases that destabilize *S. aureus* biofilms, reducing pathogen colonization [39]. On the same note, *C.* *accolens* releases free fatty acids, which prevent the exacerbation of pneumococcal disease and ensure a balance of the epithelial status [42]. These mutually beneficial defenses are an example of the defensive roles of the commensal community. Humidity, temperature, exposure to particulate matter, and smoking are environmental and behavioral factors that play a significant role in influencing microbial composition [44]. The reduction in beneficial taxa, such as *Dolosigranulum*, and an increase in pathogenic genera are caused by pollutants and cigarette smoke [45]. The use of antibiotics and food habits also contribute to the microbial balance of the nose, and thus, they are signals of both local and global well-being [46].

**Figure 1:**
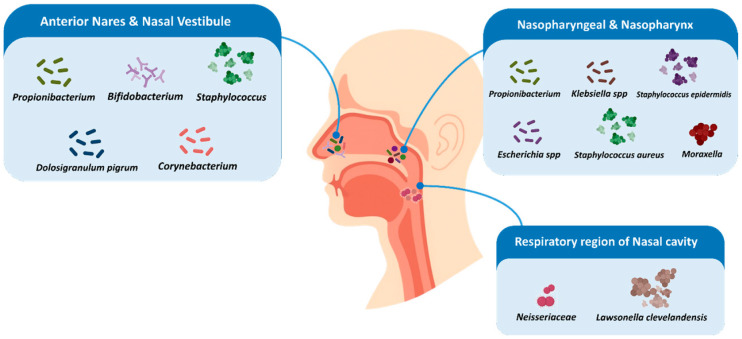
Schematic representation of the compartmentalized nasal microbiome. The anterior nares and nasal vestibule are commonly enriched in *Cutibacterium/Propionibacterium*, *Bifidobacterium*, *Staphylococcus* spp., *Dolosigranulum pigrum*, and *Corynebacterium* [30,31,33,35,36,37,38]. The nasopharynx may harbor *Cutibacterium/Propionibacterium*, *Klebsiella* spp., *Staphylococcus epidermidis*, *Escherichia* spp., *Staphylococcus aureus*, and *Moraxella* spp., particularly in inflammatory or infectious contexts [30,32,33,39,40]. The respiratory region of the nasal cavity includes members of the *Neisseriaceae* family and *Lawsonella clevelandensis*, for which olfactory relevance is currently supported by limited evidence [37,41].

The visual representation of the topographical distribution of the microbial population amongst the nasal compartments is shown in Figure 1. The nasal cavity has large populations of defensive commensals, including *S. epidermidis* and *Corynebacterium*; the nasopharynx has a higher population of opportunistic pathogens, including *Moraxella* and *Haemophilus* [28,29,47,48,49]. This geographical separation, as shown in the figure, points to the exclusive microbial gradients of the nasal cavity and their possible functional implications on mucosal immunity and olfactory sensitivity [50].

## 3. Olfactory Regulation Mechanisms

OE is a specialized neuroepithelial cell layer, which has sensory neurons, sustentacular cells, and basal progenitors that permit the constant renewal of neurons [51]. Microbes that are in the proximity of the OE are capable of informing olfactory sensitivity through mediating immune and epithelial responses.

Important signaling molecules, which also control tight-junction integrity and odorant receptor gene expression, are microbial metabolites (especially SCFAs such as butyrate and acetate) [52]. Bifidobacterial activity ensures the integrity and stability of mucus and supports neuronal regeneration, whereas dysbiosis leads to the release of inflammatory cytokines that suppress receptor activity and neural signal transmission [45]. Pattern-recognition receptors (PRRs) such as TLR2, TLR4, and TLR9 are expressed on the nasal epithelium, and detect microbial-associated molecular patterns [53]. Homeostatic stimulation of these receptors facilitates the repair of the mucosa, but chronic stimulation through pathogenic organisms leads to chronic inflammation and sensory impairment [54].

Olfactory neuron renewal and signaling can further be disrupted by pro-inflammatory cytokines like IL-6, TNF-α, and IFN-γ [55]. Therefore, the nasal microbiome’s connections with immune signaling and olfactory neurons are a complex regulatory system that defines sensory health.

### 3.1. Signal Transduction

Signal transduction in the nasal mucosa entails the interaction of microbial metabolites, epithelial cells, and the local immune system [56]. The nasal microbiome has been found to affect host signaling through metabolites like SCFAs, indoles, and other microbial products that control epithelial gene expression and barrier activity [57].

Metabolites bind to G protein-coupled receptors (GPCRs) and nuclear receptors in epithelial and immune cells and regulate local cytokine production and receptor sensitivity [58]. As an example, butyrate improves epithelial tight-junction integrity and decreases inflammatory signals by suppressing histone deacetylases, thus increasing tissue stability [59].

On the contrary, bacterial toxins and pathogen-associated molecular patterns (PAMPs) like lipopolysaccharides (LPS) activate TLR2 and TLR4, which trigger the pathway of NF-kB-regulated transcription of pro-inflammatory genes [46,60]. Excessive stimulation of these mechanisms leads to the overproduction of cytokines (e.g., IL-6, TNF-α, and IL-8), the apoptosis of the endothelium, and the impairment of the regeneration of olfactory neurons [51,61].

It is the reciprocal interaction between microbial-associated signals and host transduction responses that thus determines whether immune tolerance or inflammation prevails. Regulated microbial signaling promotes mucosal homeostasis, and uncontrolled activation is the basis of chronic inflammation and loss of sensory functions [62,63].

### 3.2. Microbiome-Driven Diversity

The biodiversity of microbes is a very important factor in nasal ecosystem resilience. A heterogeneous community of microbes has been shown to offer functional redundancy that averts pathogenic overgrowth and balances the mucosa [64]. In healthy persons, healthy taxa (*C. accolens* and *D. pigrum*) co-exist with commensal Staphylococcus species, which play a role in immunological homeostasis and epithelial fitness [65].

A decrease in microbial diversity is associated with inflammation and olfactory loss and is observed in various conditions, such as CRS, allergic rhinitis, virus infection, etc. [66]. The loss of protective commensals causes local metabolic disturbance and the growth of opportunistic pathogens, including *S. aureus* and *P. aeruginosa* [50]. The nasal microbial diversity is also defined by environmental factors such as air pollution, work-related exposures, and use of antibiotics [67]. Pollutants cause oxidative stress and alter the pH of the mucosal surface, which favors pro-inflammatory microbial species [55]. On the same note, antibiotics can temporarily decrease the microbial count at the expense of recolonizing beneficial species, facilitating infection relapses [56].

Hence, microbial diversity is central to mucosal integrity, immune tolerance, and sensory perception. Cessation of this diversity preconditions the nasal mucosa to chronic inflammation and immune system disorders.

### 3.3. The Mucosal Layer and Host Interaction

The nares’ mucosal layer is a dynamic structure, in which epithelial, microbial, and immune components enable homeostasis via continuous interaction [58]. Both physical and biochemical barriers formed by the mucus secreted through goblet cells and submucosal glands trap inhaled particles, allergens, and microorganisms [59].

Commensal bacteria play a role in promoting the health of the mucosa through the formation of AMPs, mucin-degrading enzymes that maintain the viscosity of mucus [46]. They also regulate signaling in epithelial cells, which affects tight-junction integrity and expression of cytokines [64]. Pathogenic species interfere with this balance during dysbiosis. Both overproduction of mucus and the development of microbial biofilms result in the inhibition of ciliary clearance and long-term infection [65]. The biofilms that *S. aureus* and *P. aeruginosa* develop are resistant to host immune responses, which continues to cause inflammation and tissue remodeling [66].

The epithelial tissue is also an immunologically active structure. It releases cytokines and AMPs, including β-defensins and lysozymes, upon response to microbial stimuli [53]. These molecules are not only responsible for regulating the colonization of bacteria, but also the health of olfactory neurons and sensory transduction. Therefore, mucosal–microbial interactions are a protective and regulatory response, which combine microbial and host communication to preserve OF [67].

### 3.4. Immune Mechanisms in the Nasal Microbiome

#### 3.4.1. Innate Immunity

The first line of defense against microbial imbalance of the nasal cavity is innate immunity [68]. PRRs, including TLR2, TLR4, and NOD-like receptors (NLRs), are used by epithelial and immune cells to recognize microbial components [31]. The receptors sense bacterial cell wall constituents and trigger downstream signaling cascades, which activate NF-kB and other transcription factors [57].

These pathways are regulated to ensure epithelial integrity and repair. Nevertheless, chronic stimulation by pathogenic bacteria causes chronic production of cytokines (IL-1β, IL-8, and TNF-α) and neutrophil and macrophage recruitment, which is capable of damaging the surrounding tissues [58].

Defensins and cathelicidins are ectodermal-origin AMPs that play an important part in innate immunity, restricting pathogen colonization and promoting commensal stability [59]. When microbial diversity is in balance with the innate immune regulation, it guarantees the protection of the mucosal system as well as the prevention of excessive inflammation, which can hamper olfactory neurons [51].

#### 3.4.2. Adaptive Immunity

T and B lymphocytes play a critical role in long-term mucosal homeostasis by having an adaptive immune response [61]. Secretory immunoglobulin A (sIgA), synthesized by the plasma cells of the submucosa, neutralizes pathogens and inhibits the adhesion of the microbes. A decrease in the level of sIgA is linked with microbial overgrowth and chronic inflammation, as was found in CRS and allergic rhinitis [63]. Subsets of T helper cells, such as Th1, Th2, and Th17, mediate the release of cytokines that characterize the inflammatory microenvironment [64]. T17 cells stimulate neutrophilic inflammation, and Tregs are regulatory T cells that prevent the overstimulation of the immune system and tolerance [52]. The loss of the Th17/Treg balance may lead to long-term inflammation and tissue destruction [65].

This immune deregulation is not only involved in the progression of nasal disease but also in the renewal of olfactory neurons, which results in permanent sensory impairments [66]. Table 2 summarizes the main immune mediators and specific relationships between them and the nasal microbiome. The following table summarizes both innate and adaptive parts and highlights their two functions in defense and regulation. Secretory IgA preserves the normal microbial balance by stopping the adhesion of pathogens and enhancing immune tolerance [58]. The local inflammatory balance is orchestrated by defensins and cytokines such as IL-1β and TNF-α, and the recognition of microbial motifs and the subsequent induction of the immune response are mediated by TLRs [59].

Abnormal regulation of such pathways in CRS and allergic rhinitis usually leads to excessive cytokine secretion, neutrophil inflammation, and loss of barrier function. The summarized data presented herein demonstrate that microbial signals may alter immune responses from protective homeostasis to chronic inflammation, which highlights the primary centrality of microbe–immune system crosstalk in olfactory and respiratory pathology.

### 3.5. Nasal Microbiome in Contexts of Disease

#### 3.5.1. Chronic Rhinosinusitis (CRS)

CRS is one of the most widely researched disorders that connect nasal microbiome and OD. It is defined by chronic inflammation of the mucosa, biofilm development, and dysbiosis [53]. Research studies have always found depleted levels of *Corynebacterium* and *Dolosigranulum*, as well as a growth in the number of *S. aureus* and *P. aeruginosa* in CRS patients [54]. The microbial changes that can be summarized in Table 1 are in line with CRS-related dysbiosis, where commensal loss and opportunistic pathogen gain are continuous contributors of mucosal inflammation and olfactory loss [54].

The pathogenic dominance will result in augmentation of cytokine synthesis and epithelial barrier breakdown, specifically through TLR2/TLR4 signaling pathways [55]. Persistent exposure to inflammatory products like IL-6 and TNF-α also worsens the situation by damaging the olfactory neurons, which can regenerate and perform their functions [68].

The treatment prospects of microbial repair with the help of probiotics and topical care are promising to alleviate inflammation and enhance olfactory results [69]. Commensal restoration has the potential to suppress biofilm development as well as restore immune balance [57].

#### 3.5.2. Allergic Rhinitis and Environmental Factors

Allergic rhinitis is an exaggerated Th2-type reaction with increased levels of IgE and eosinophilic inflammation [58]. Changes in the nasal microbiome in allergic rhinitis are commonly associated with losses in diversity and gains in Staphylococcus species, which stimulate mucosal inflammation and defective barrier operations [59]. This imbalance is worsened by environmental contaminants, allergens, and smoking, which cause oxidative stress and change epithelial cytokine signaling [56].

The changes not only contribute to long-term inflammation, but also to the olfactory impairment that is typical of patients with allergic rhinitis [39]. Microbial stability recovery by specific interventions can help prevent allergic inflammation and maintain olfactory capability.

### 3.6. Viral Infections and Olfactory Dysfunction

One of the most common and clinically relevant causes of the loss of OF is a viral infection. As the main entry point of the virus, the nasal mucosa serves as the entry point of multiple respiratory pathogens that can destabilize epithelial integrity, provoke inflammation, and change the microbiome that dwells in the nasal cavity. Cellular apoptosis, cytokine release, and secondary bacterial colonization as a result of viral replication in the nasal and OE impair the process of detecting odors, as well as transmission of signals [67]. The nature and duration of the olfactory loss are determined by the nature of the virus, the host immune response, and the extent of the epithelial and neuronal injury [51].

Various respiratory viruses, specifically coronaviruses, influenza, and parainfluenza, disrupt the sustentacular cells, as well as olfactory sensory neurons, triggering local inflammation and consequent failure to maintain the expression of odorant receptors. Moreover, the release of cytokines caused by viruses changes the nasal microbial composition to an acute dysbiosis that can last beyond the elimination of viruses [61]. The combination of viral pathogen-related immune activation and microbiome imbalance underlies post-infectious olfactory disorders.

#### 3.6.1. SARS-CoV-2

The global COVID-19 pandemic provided a unique opportunity to investigate the mechanisms underlying SARS-CoV-2-associated anosmia. SARS-CoV-2 enters into sustentacular and glandular epithelial cells that express the ACE2 receptor and the serine protease TMPRSS2, triggering a localized inflammatory reaction and epithelial shedding [57]. However, unlike previous coronaviruses, SARS-CoV-2 seems to attack most olfactory neurons sparingly, but its cytopathic impact on supporting cells alters the ionic and metabolic microenvironment that the neurons need in order to function [58]. The resultant upsurge of cytokines, such as IL-6, TNF-α, and interferons, impairs the integrity of the epithelia and slows down the regeneration of sensory neurons.

OD is an early symptom of SARS-CoV-2 infection and may precede respiratory manifestations, representing a useful clinical indicator of viral involvement in the upper airway. Although the majority of people are able to regain their smell within weeks, a major part experience long-term anosmia or hyposmia, which is the manifestation of long-lasting inflammation of the epithelia, destruction of microvasculature, and failure of stem-cell-mediated recovery [59]. Recent studies indicate that nasal dysbiosis—characterized by reduced commensal diversity and expansion of opportunistic taxa—may persist after SARS-CoV-2 clearance, sustaining inflammatory responses despite viral elimination [46]. Microbial homeostasis by probiotic or microbiome-modulating therapies has thus been suggested as a complementary treatment modality to speed up mucosal recovery and olfactory recuperation.

#### 3.6.2. Other Viral and Post-Infectious Olfactory Loss

In addition to SARS-CoV-2, many widespread respiratory viruses cause OD, such as influenza, parainfluenza, rhinovirus, and respiratory syncytial virus, which damage the structures of epithelial and neuronal cells [51]. Viral replication causes the apoptosis of sustentacular and basal cells, and the host immune response that accompanies it produces a large amount of reactive oxygen species (ROS), worsening the tissue damage [61]. Acute viral attack is, in most instances, preceded by chronic inflammatory alterations and subsequent bacterial colonization of the mucosa, which further disrupts mucosal homeostasis.

Maintenance of olfactory loss following the infection is an indication of a multifactorial mechanism that entails oxidative stress, immune dysregulation, and microbiome imbalance. Sustained stimulation of the local immune response impairs the recovery of the olfactory receptor neurons and maintains tissue remodeling mediated by cytokines. Additionally, the loss of favorable taxa, including *Corynebacterium* and *Dolosigranulum*, can cause a decrease in mucosal resilience and slow repair of the epithelium. Awareness of this interaction between viral infection, immune response, and microbial disruption is important to therapeutic approaches focusing on both the inflammation and microbiome restoration [62,63]. Combinations of anti-inflammatory therapy and specific microbial re-equilibration have led to promising improvements in outcomes of recovery and prevention of persistent post-infectious olfactory loss.

### 3.7. Nasal Decolonization Protocols and Microbiome Modulation

Decolonization is also focused on restoring a microbial balance that is selective in reducing the pathogenic load but does not affect commensal stability [52]. Traditional antiseptic solutions, including mupirocin and povidone-iodine, are effective against *S. aureus* but can destroy beneficial microbes in the case of excessive use [65].

Other interventions, such as probiotic-based interventions, bacteriophage therapy, and nasal saline irrigation are under active consideration to foster microbial homeostasis [66]. *Lactobacillus sakei* and *Lactobacillus plantarum* have demonstrated the potential to inhibit pathogens and the regulation of mucosal immunity [50].

The microbial resilience can also be enhanced with preventive measures like staying hydrated, eliminating pollutants, and ensuring optimal nutrition [67]. Altogether, it is possible to note that nasal microbiome modulation is a promising adjunctive method of treating olfactory and inflammatory conditions [68].

## 4. Neuropathological Correlates of Nasal Dysbiosis

The OE represents a unique anatomical and physiological interface between the external environment and the central nervous system [1,3]. In recent years, nasal dysbiosis has been increasingly associated with markers of neuroinflammation, protein aggregation, and neuronal vulnerability. However, current evidence, largely derived from preclinical and experimental models, does not establish a direct causal relationship, instead supporting a hypothesis-generating link between nasal microbial imbalance and neurological health [5,70]. Accumulated data, primarily associative in nature, suggest that microbial metabolites, pro-inflammatory cytokines, and impaired mucosal immunity may be linked to neuropathological processes relevant to PD and AD, potentially modulating disease-related pathways rather than directly initiating them [6,7].

Sustained nasal inflammation has been proposed to facilitate changes in the translocation of inflammatory mediators and microbial components along olfactory pathways, potentially influencing microglial activation and neuronal signal transduction within the olfactory bulb and connected brain regions [8,9]. In this context, microbial-derived amyloid-like proteins and lipopolysaccharides may act as permissive factors by promoting protein misfolding or amplifying neuroinflammatory signaling. Experimental studies have shown that bacterial amyloids can function as cross-seeding templates, facilitating the aggregation of neurodegeneration-related proteins such as α-synuclein and tau [10,71].

α-synuclein and tau are neuronal proteins that, under pathological conditions, undergo misfolding and aggregation, leading to synaptic dysfunction and neuronal loss [15,72]. In PD, α-synuclein aggregates to form Lewy bodies, whereas in AD, tau aggregation results in neurofibrillary tangles [16]. Experimental and observational evidence suggests that α-synuclein pathology may involve the olfactory epithelium as an early site of involvement; however, this model remains speculative and is largely supported by animal studies and post-mortem observations [17,18]. This hypothesis is consistent with clinical observations indicating that olfactory dysfunction often precedes motor symptoms by several years in PD [19].

Microbial-derived amyloids, inflammatory mediators, and oxidative stress have been shown to accelerate α-synuclein and tau aggregation and to enhance neuronal susceptibility to misfolding-related damage [20,73]. For example, curli fibers produced by *Escherichia coli* have been reported to cross-seed α-synuclein aggregation, thereby promoting neuroinflammatory responses in experimental models [21,22]. In parallel, microglial activation induced by lipopolysaccharides and other bacterial components may further contribute to neuronal vulnerability through sustained inflammatory signaling and redox imbalance [24,25].

Although a reduction in olfactory sensory neurons might intuitively be expected to limit pathogen access to the central nervous system, neuronal loss in inflammatory conditions is typically accompanied by a broader collapse of olfactory epithelial barrier function. Inflammatory damage to sustentacular cells, disruption of tight junctions, and degradation of extracellular matrix components compromise both the structural and immunological integrity of the olfactory epithelium, resulting in increased epithelial permeability [44,74].

This barrier dysfunction facilitates the translocation of microbial products, cytokines, and other inflammatory mediators toward the olfactory bulb, despite partial neuronal depletion [8,9,46,75]. In parallel, epithelial injury and cytokine-driven inflammation may further weaken blood–brain barrier integrity, amplifying central nervous system exposure to peripheral inflammatory signals [65,67]. This barrier-centric mechanism helps reconcile neuronal loss with an increased susceptibility to pathogen- or inflammation-mediated brain involvement.

Collectively, these observations support the view that nasal dysbiosis may represent a permissive or upstream factor associated with neurodegenerative proteopathies, rather than a direct causal driver. While alterations in the nasal microbiome and chronic mucosal inflammation may contribute to pathways relevant to neurodegeneration, direct causality in human populations has not been demonstrated [11,13]. As such, these mechanisms should be regarded as hypothesis-generating, underscoring the need for longitudinal human studies and targeted experimental models to clarify temporal relationships and clinical relevance (Supplementary Table S1).

### Cytokine-Mediated Mechanisms of Olfactory Neurodegeneration

Pro-inflammatory cytokines released during chronic nasal inflammation, including IL-1β, IL-6, and TNF-α, have been implicated in mechanisms that may contribute to olfactory neurodegeneration. These mediators can disrupt epithelial barrier integrity and alter the metabolic and ionic support provided by sustentacular cells, thereby compromising the microenvironment required for olfactory sensory neuron survival [29].

In addition, cytokine diffusion along olfactory pathways may promote microglial activation within the olfactory bulb, leading to sustained neuroinflammatory signaling and increased production of reactive oxygen species [30,33]. Prolonged oxidative stress can damage neuronal membranes, mitochondria, and cytoskeletal structures, ultimately increasing neuronal vulnerability [43].

Importantly, excessive cytokine signaling has also been associated with impaired regeneration of olfactory sensory neurons, as inflammatory mediators interfere with stem-cell-mediated renewal processes within the olfactory epithelium [66,68]. Together, these findings suggest that cytokine-driven inflammation may contribute to olfactory neurodegeneration through combined effects on neuroinflammation, oxidative damage, and failed neuronal repair, while remaining primarily supported by experimental and associative evidence.

## 5. Discussion

### 5.1. Integrative Mechanisms Linking Microbiome and Olfactory Dysfunction

The interaction between the nasal microbiome, immune responses, and neuronal activity is increasingly recognized as a key determinant of olfactory health [45]. Rather than acting through isolated pathways, microbial imbalance appears to influence olfactory function indirectly by modulating immune tone, epithelial resilience, and inflammatory signaling, including alterations in microbial metabolite production such as short-chain fatty acids (SCFAs) [57], as well as inflammation-driven effects on epithelial regeneration and local immune responses [53].

Clinical conditions such as chronic rhinosinusitis and viral infections exemplify contexts in which microbial dysbiosis is associated with local and systemic inflammation and sensory impairment [50,54]. Similarly, long-term exposure to environmental pollutants or allergens may alter mucosal immunity and influence microbiome composition, thereby increasing susceptibility to olfactory dysfunction [36]. Importantly, these conditions should be interpreted as models of interaction rather than linear cause–effect relationships.

The compartmentalized organization of the nasal microbiome, as illustrated in Figure 1, may further contribute to region-specific immune regulation, potentially influencing the differential vulnerability of the olfactory epithelium to infectious or inflammatory insults [45,66].

Finally, emerging evidence supports a bidirectional relationship between the nasal microbiome and the central nervous system, whereby microbial products may influence brain activity through immune and neuroendocrine mechanisms, while neurodegenerative processes may in turn alter mucosal immunity and microbial colonization patterns [55,68]. Together, these observations highlight the dual role of the nasal cavity as a sensory and immunological interface, where microbial, immune, and neural networks converge in health and disease [56,69].

### 5.2. Methodological and Research Limitations

Within the body of evidence examined in this manuscript, investigations linking nasal microbiome alterations to neurodegenerative diseases are predominantly cross-sectional or based on preclinical models. Consequently, these findings should be interpreted as associative rather than causal, highlighting the need for longitudinal human studies to clarify temporal relationships and underlying mechanistic pathways.

Although improving quickly, there are still major methodological issues with the investigation of the nasal microbiome. Microbial community profiles can all be affected by sampling variability, DNA extraction procedures, and sequencing biases [58]. The complexity of the anatomy of the nasal cavity, with a variety of microenvironments and low microbial biomass, also makes it difficult to adequately characterize the nasal cavity [59]. Longitudinal plans with metagenomics, transcriptomics, and metabolomics need to be conducted to help shed light on the effects of particular taxa and metabolites in shaping olfactory and neurological outcomes [57,69].

Sampling methods and workflows should undergo standardization to increase cross-study reproducibility and comparability. Moreover, it is possible to combine clinical data with molecular analyses in order to translate microbial signatures into diagnostic or therapeutic uses [46,60].

### 5.3. Clinical and Translational Implications

A clearer understanding of the nasal microbiome may support the future exploration of novel strategies for addressing olfactory and neurological disorders, rather than immediate clinical application [51,61]. Microbiome-targeted approaches, including probiotics, prebiotics, and bacteriophage-based modalities, have been proposed as potential means to modulate microbial balance and mucosal inflammation, although their clinical efficacy remains to be established [62,63].

Restoring balanced interactions between commensal microorganisms and key immune components represents a conceptual framework for intervention, as summarized in Table 2. In this context, selected probiotic strains reported to influence secretory IgA levels or Toll-like receptor signaling have been explored for their capacity to modulate inflammation and support the recovery of olfactory function in experimental or early clinical settings [52,64]. Topical application of commensal strains, including *Lactobacillus sakei* and *Lactobacillus plantarum*, has shown preliminary potential in regulating mucosal immunity and improving olfactory outcomes, although supporting evidence remains limited [64].

Beyond local olfactory disorders, microbiome modulation has been hypothesized to hold relevance for neurodegenerative diseases in which olfactory impairment represents an early or accompanying feature, rather than a confirmed preventive strategy [52,65].

At the clinical level, nasal microbiome profiling may emerge as a complementary biomarker to assess disease risk or support the identification of neurodegenerative conditions [45,66]. From a translational perspective, the nasal cavity can therefore be viewed as an interface at the intersection of immunology, neurology, and microbiology, with potential diagnostic and therapeutic relevance that warrants further investigation [50,54].

### 5.4. Future Perspectives

Future research should focus on elucidating the molecular communication between the nasal microbiome and the host nervous system. In particular, integrated omics approaches, including genomics, metabolomics, and proteomics, may enable the investigation of microbial metabolites and signaling pathways relevant to olfactory function and neuroimmune interactions [55,68].

Mechanistic insights may be further advanced through the development of physiologically relevant in vitro models, such as nasal organoids and co-culture systems incorporating epithelial, immune, and neuronal components [31]. In parallel, host–microbiome–neuron interactions and immune modulation can be explored using in vivo models, including *Galleria mellonella* and murine systems, which offer complementary experimental advantages [58,69].

Finally, personalized medicine approaches that integrate individual microbiome profiles, genetic susceptibility, and environmental exposures may inform the future development of more selective and targeted interventions [49,59]. Achieving this goal will require enhanced interdisciplinary collaboration among microbiologists, clinicians, and neuroscientists to facilitate translational progress.

## 6. Conclusions

The nasal microbiome represents a biologically active component of the olfactory system that interacts with epithelial integrity, immune regulation, and neuronal function. Alterations in microbial balance may be associated with inflammatory mechanisms that extend beyond the nasal cavity and are relevant to broader neuroimmune and neurodegenerative pathways.

Rather than acting as a passive reservoir, the nasal microbiome appears to participate in dynamic host–microbe interactions with potential implications for olfactory dysfunction and related neurological conditions. However, current evidence remains largely associative, and the translational relevance of these findings is yet to be established.

Future research should prioritize longitudinal human studies, mechanistic investigations, and integrative multi-omics approaches to clarify causal relationships and define the role of the nasal microbiome within the nasal–brain axis.

## Figures and Tables

**Table 1:** Summary of nasal microbiome strains, anatomical locations, clinical and olfactory relevance.

Strains	Presence	Clinical and Olfactory Relevance	References
***Staphylococcus aureus***	Epithelial Layer—Anterior Nares (nostrils)	Opportunistic pathogen associated with nasal and respiratory infections and antibiotic resistance; indirectly associated with olfactory dysfunction in infectious or inflammatory conditions	[32,33,34]
***Staphylococcus epidermidis***	Epithelial Layer—Anterior Nares (nostrils)	Common nasal commensal associated with microbial balance and mucosal homeostasis; protective associations reported in observational studies	[33]
***Corynebacterium* spp.**	Epithelial Layer—Nasal Vestibule	Common nasal commensal associated with microbial balance and mucosal homeostasis; protective associations reported in observational studies.	[30,33,35,36]
***Dolosigranulum pigrum***	Epithelial Layer—Nasal Vestibule	Commensal taxon associated with respiratory health and microbial stability; potential indirect relevance to olfactory function	[33,37,38]
***Moraxella* spp.**	Epithelial Layer—Nasopharynx (upper part of the throat)	Associated with respiratory infections and otitis media; olfactory impairment may occur secondary to inflammatory respiratory disease	[30]
***Bifidobacterium* spp.**	Epithelial Layer—Nasal Cavity	Potential probiotic candidate, beneficial for respiratory health, may support respiratory health and indirectly influence olfactory function.	[31]
***Cutibacterium* (*Propionibacterium*) spp.**	Epithelial Layer—Nasal Cavity	Skin-associated commensals also detected in the nasal cavity; limited and indirect evidence regarding olfactory relevance	[30]
***Escherichia* spp.**	Epithelial Layer—nasopharyngeal region of nasal cavity	Indicator of gut microbiome health and potential pathogen may impact olfactory function due to gut microbiome health.	[39]
***Klebsiella* spp.**	Epithelial Layer—nasopharyngeal region of the nasal cavity	Opportunistic pathogens associated with respiratory infections; potential indirect effects on olfactory function during infection	[40]
***Lawsonella clevelandensis***	Nasal cavity—Respiratory region	Emerging pathogen reported in respiratory conditions; olfactory relevance remains poorly characterized	[37,41]
***Neisseriaceae* (family)**	Nasal cavity—Respiratory region	Component of the upper respiratory microbiome; proposed indicator of mucosal ecological state with limited data on olfactory impact	[37,41]

**Table 2:** Immune components of the nasal mucosa and their microbial interactions. This table summarizes key immune factors, their roles in maintaining nasal health, disease-associated dysregulation, and interactions with microbial elements.

Immune Component	Role in Health	Dysregulation in Disease	Microbial Influence	References
**Secretory IgA (sIgA)**	Pathogen defense, microbiome stability	Reduced in CRS	Limits bacterial colonization	[58]
**Defensins**	Innate immune response, microbial regulation	Altered expression in viral infections	Protects against pathogens	[49,59]
**Cytokines (e.g., IL-1β, TNF-α)**	Immune signaling, inflammation control	Elevated in CRS and allergic rhinitis	Modulates immune response to microbes	[49,69]
**Toll-like receptors (TLRs)**	Pathogen recognition, immune activation	Dysregulation in respiratory infections	Detects microbial components	[49]
**Neutrophils**	Phagocytosis, pathogen clearance	Increase in bacterial infections	Responds to microbial presence	[49]

## Data Availability

No new data were created or analyzed in this study. Data sharing is not applicable to this article.

## Supplementary Materials

The following supporting information can be downloaded at https://www.mdpi.com/article/10.3390/microorganisms14010234/s1, Supplementary Table S1. Mechanistic overview of nasal dysbiosis and neuro-olfactory dysfunction.

## Abbreviations

The following abbreviations are used in this manuscript:

AD	Alzheimer’s disease
AMP	Antimicrobial peptide
BBB	Blood–brain barrier
IL-1β	Interleukin-1 beta
CRS	Chronic rhinosinusitis
GPCR	G protein-coupled receptor
LPS	Lipopolysaccharides
NLR	NOD-like receptor
OB	Olfactory bulb
OE	Olfactory epithelium
OF	Olfactory functions
OD	Olfactory dysfunction
PD	Parkinson’s disease
PRR	Pattern-recognition receptor
ROS	Reactive oxygen species
sIgA	Secretory immunoglobulin A
SCFA	Short-chain fatty acid
TNF-α	Tumor necrosis factor-alpha
TLRs	Toll-like receptors
