# Supplementary material for: Clinical and Immunological Perspectives on the Nasal Microbiome’s Role in Olfactory Function and Dysfunction
Source: Microorganisms. 2026 Jan 20;14(1):234. doi: 10.3390/microorganisms14010234 (PMC12843637; doi:10.3390/microorganisms14010234)
Supplement: Supplementary file 1 [file microorganisms-14-00234-s001.zip › microorganisms-4082600-supplementary.pdf]

Supplementary Table S1. Mechanistic overview of nasal dysbiosis and neuro-olfactory dysfunction

Core Mechanism	Mechanistic Driver	Biological Consequence	Pathway Type	Impact on Neuro-Olfactory Function	References
OE–CNS anatomical and inflammatory access	Direct anatomical interface between olfactory epithelium and CNS combined with local inflammation	Increased exposure of central nervous system to nasal microbial and inflammatory signals	Anatomical / Translocation	Increased susceptibility to neuroinflammatory signaling	[1,3,8,9]
Chronic neuroinflammation	Dysbiosis-driven activation of inflammatory cytokines (e.g., IL-1β, IL-6, TNF-α)	Sustained glial activation and neuronal stress	Inflammatory	Increased risk of chronic neuroinflammation	[5,49,70]
Microbial metabolites and proteopathic stress	Microbial amyloids, lipopolysaccharides, and pathogenic metabolites	Promotion of α-synuclein and tau misfolding and aggregation	Proteopathic	Increased burden of misfolded proteins	[7,10,34,71]
Oxidative and cellular stress	Reactive oxygen species accumulation and immune dysregulation	Structural neuronal damage	Oxidative	Increased neuronal vulnerability	[43]
Epithelial and BBB dysfunction	Weakening of olfactory epithelial and blood–brain barriers	Enhanced entry of toxins and cytokines into the brain	Barrier	Increased CNS exposure to inflammatory mediators	[46,65,67,75]
Early associative neurodegenerative processes	Persistent nasal dysbiosis and chronic mucosal inflammation	Association with PD- and AD-related molecular pathways	Associative	Early neuropathological correlates	[11,13,62,63]

This table summarizes the principal mechanisms linking nasal microbial imbalance to olfactory and neurological alterations. Mechanisms have been consolidated to reduce redundancy and reflect primarily associative or experimental evidence rather than established causality.
